# Integration the biologic factors into the staging of breast cancer patients with ipsilateral supraclavicular lymph node metastasis

**DOI:** 10.7150/jca.52449

**Published:** 2020-09-30

**Authors:** Chen-Lu Lian, Xiao-Yang Cai, Ping Zhou, Jun Wang, Xiu-Bei Chen, San-Gang Wu

**Affiliations:** 1 Department of Radiation Oncology, the First Affiliated Hospital of Xiamen University, Xiamen 361003, People's Republic of China.; 2 Department of Intensive Care Unit, the First Affiliated Hospital of Xiamen University, Xiamen 361003, People's Republic of China.

**Keywords:** breast cancer, ipsilateral supraclavicular lymph nodes, staging, prognosis, AJCC

## Abstract

**Purpose:** To investigate the accuracy and the discriminatory performance in the prognostic prediction in breast cancer (BC) patients with ipsilateral supraclavicular lymph node (ISLN) metastasis using the between the American Joint Committee on Cancer (AJCC) 7th and 8th edition staging system.

**Methods:** Female patients diagnosed as BC were retrieved from the Surveillance, Epidemiology, and End Results database between 2010 and 2014. Chi-squared test, Kaplan-Meier method, Cox proportional hazard analysis, and the receiver operating characteristics were used to conduct statistical analysis.

**Results:** We included 1097 BC patients with ISLN metastasis (N3c disease), including 29.4% (n=322) and 70.6% (n=775) of patients with non-metastatic and metastatic stage at diagnosis, respectively. In non-metastatic stage patients, 64.9% of the patients categorized as having stage IIIC disease in the 7th edition AJCC staging system were downstaged to stage IIIA or IIIB according to the 8th AJCC staging criteria. The AJCC 8th edition staging system had better discriminatory prognostic value than the 7th AJCC staging (area under the curve: 0.586 vs. 0.577, *P*=0.0006), with a 5-year breast cancer-specific survival (BCSS) rate of 71.3%, 62.2%, 45.2% and 39.1% in stage IIIA, IIIB, IIIC, and IV cohorts, respectively (*P*<0.0001). The multivariate prognostic analysis revealed that the AJCC 8th edition staging system was an independent prognostic factor for BCSS, while no statistical difference in BCSS was found between the 8th AJCC stage IIIC and IV patients (*P*=0.188).

**Conclusion:** The AJCC 8th edition pathological prognostic staging showed a better discriminatory prognostic value in ISLN-metastasized breast cancer patients. An additional clarification strategy in stage IIIC disease based on the 8th AJCC staging should be developed to differentiate patients who are curable with multimodality therapy and patients who have less benefit from curative treatment.

## Introduction

Breast cancer (BC), the most commonly diagnosed female cancer, is also the leading cause of women cancer-related death in most regions and countries of the globe 1. The incidence of ipsilateral supraclavicular lymph node (ISLN) metastasis in BC patients without distant metastasis accounted for a small proportion of 1-4.3% 2. Historically, it was generally believed that ISLN-metastasized BC was a locoregional disease and should be assigned to stage IIIB 3. It was not until 1988 that ISLN-metastasized BC was defined as stage M1 disease in the 3rd American Joint Committee on Cancer (AJCC) Tumor-Node-Metastasis (TNM) staging system for BC due to its poor prognosis and high incidence of distant metastasis within one year 2. However, in 2002, Brito* et al.* 3 reported significantly statistical differences in prognosis between ISLN-metastasized and* de novo* stage IV disease. They believed ISLN-metastasized patients without distant metastasis should be reclassified into stage IIIB instead of stage IV. Therefore, ISLN-metastasized patients without distant metastasis have been identified as stage IIIC (N3c) disease since the 6th AJCC staging system was published 4.

In the 8th AJCC staging manual, ISLN metastasis was still categorized as N3c. This newly proposed BC pathological prognostic staging system combined several biological factors and traditional TNM factors 5. The ISLN-metastasized BC without distant metastasis was reclassified into stage IIIA, IIIB, or IIIC according to its biological characteristics in 8th AJCC criteria. However, to our knowledge, the available literature regarding the validation of prognostic prediction of the 8th AJCC staging system for ISLN-metastasized patients is rather scarce. Therefore, this retrospective study was intended to compare the accuracy and the discriminatory performance in the prognostic prediction among ISLN-metastasized patients between the 7th and 8th AJCC staging system using the Surveillance, Epidemiology, and End Results (SEER) database.

## Materials and Methods

### Patients

Female BC patients diagnosed between 2010 and 2014 were retrieved from the SEER database established by the National Cancer Institute of the United States, which collected data on demographics, clinical characteristics, the first course of treatments, and follow up for vital status (6). We included patients with T1-4N3cM0-1 breast cancer (according to the 7th AJCC-TNM staging manual). In non-metastatic stage disease (M0), the information regarding age, race/ethnicity, histology, tumor grade, T category, estrogen receptor (ER) status, progesterone receptor (PR) status, human epidermal growth factor receptor 2 (HER2) status, surgical procedure, radiotherapy and chemotherapy were available. Patients aged <18 years without positive pathological diagnoses were excluded. Since the identifiable patient information in the SEER database is de-identified, there is no need for approval from the Institutional Review Board of the First Affiliated Hospital of Xiamen University.

### Variables

The variables of this study included: age, race/ethnicity, histology, tumor grade, T category, M category, ER status, PR status, HER2 status, surgery methods, the receipt of chemotherapy and radiotherapy. The pathological prognostic stages were allocated by the AJCC 8th edition pathological prognostic staging manual, and the 7th AJCC-TNM staging manual allocated the T and M categories.

### Statistical analysis

The chi-square test or Fisher's exact test was used to compare patients' characteristics after stratification by stage change, and to perform the comparison of the characteristics among stage migration between the two editions of AJCC stages. The area under the curve (AUC) was calculated to show the discriminatory ability of the 8th AJCC staging in predicting outcomes using the receiver operating characteristics (ROC). Survival curves were drawn using the Kaplan-Meier method, and the statistical differences among stages were compared using the log-rank test. Multivariate Cox regression analysis was performed to determine the independent prognostic factors associated with breast cancer-specific survival (BCSS). All statistical analyses were performed by the IBM SPSS 26.0 software package (IBM Corp., Armonk, NY). *P* values <0.05 were considered statistical significance.

## Results

### Patient characteristics

A total of 1097 BC patients with ISLN metastasis were identified for the analysis, including 29.4% (n=322) and 70.6% (n=775) of patients with M0 stage and metastatic stage (M1), respectively. Among the stage M0 patients, 78.9% of them (n=254) had infiltrating ductal carcinoma, 50.6% (n=163) had T3-4 disease, 68.3% (n=220) had poorly/undifferentiated disease, 66.1% (n=213) had HER2-negative disease, 57.8% (n=186) had ER-positive tumors, and 42.5% (n=137) had PR-positive tumors. The majority of the stage M0 patients received multimodality treatments including surgery (n=322, 100.0%), chemotherapy (n=301, 93.5%), and radiotherapy (n=237, 73.6%). The details on patients and tumor characteristics are summarized in Table 1.

### Restaging of the AJCC 7th stage IIIC patients

Significantly statistical differences were found in the stage migration from the 7th to 8th AJCC stages (P<0.001). In the stage M0 cohort, 209 (64.9%) patients with stage IIIC diseases in the 7th staging had their stages reassigned and were downstaged into stage IIIA (n=65, 20.2%) and stage IIIB (n=144, 44.7%) according to the 8th edition criteria. Demographic and tumor characteristics of stage migration are presented in Table 2. All patients with well-differentiated (G1) diseases and 87.2% of patients with moderately differentiated (G2) diseases were downstaged, while among the poorly/undifferentiated (G3) diseases, only 54.1% were downstaged (*P* <0.001). Moreover, 86.6% and 97.1% of the ER-positive and PR-positive patients were downstaged, respectively, while only 35.3% and 41.1% of the ER-negative and PR-negative were downstaged, respectively. In addition, all patients with HER2-positive diseases were downstaged. However, only 46.9% of the HER2-negative patients were downstaged (*P* <0.001) (Table 2).

### Survival

**Figure 1:**
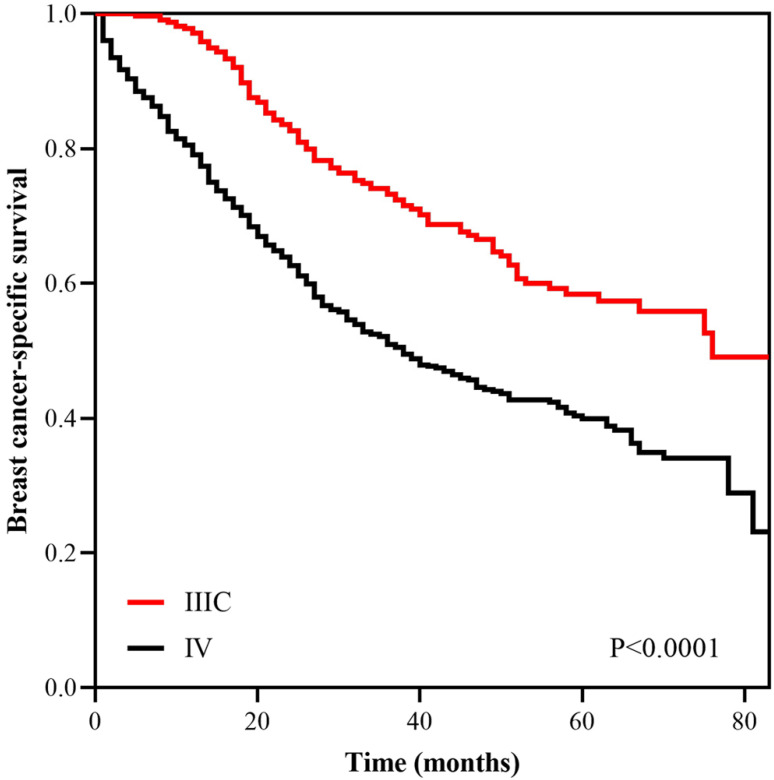
Kaplan-Meier survival curves of the AJCC 7th anatomic stage IIIC and IV patients.

**Figure 2:**
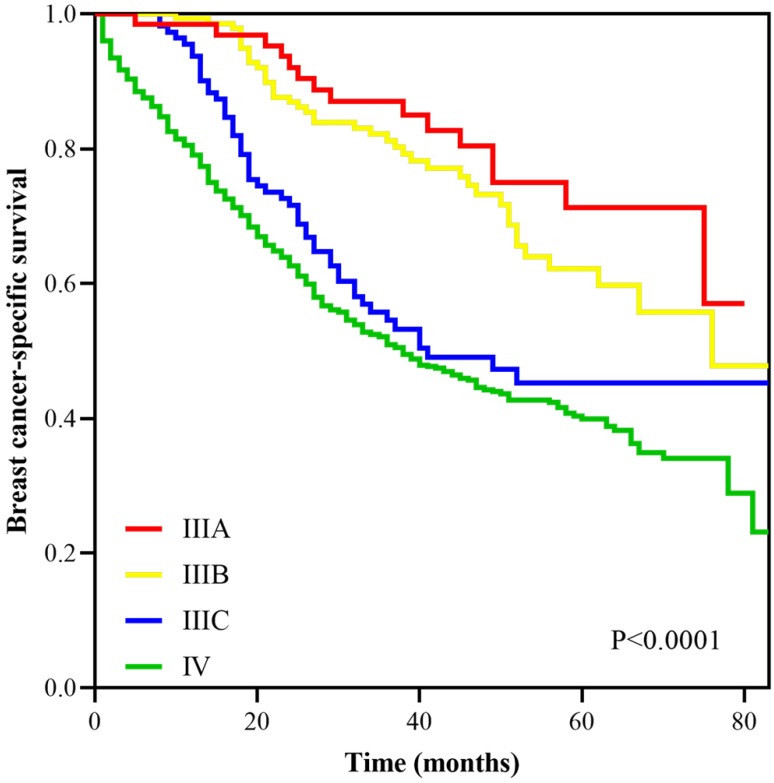
Survival curves according to different stages using the AJCC 8th pathological prognostic staging system.

**Figure 3:**
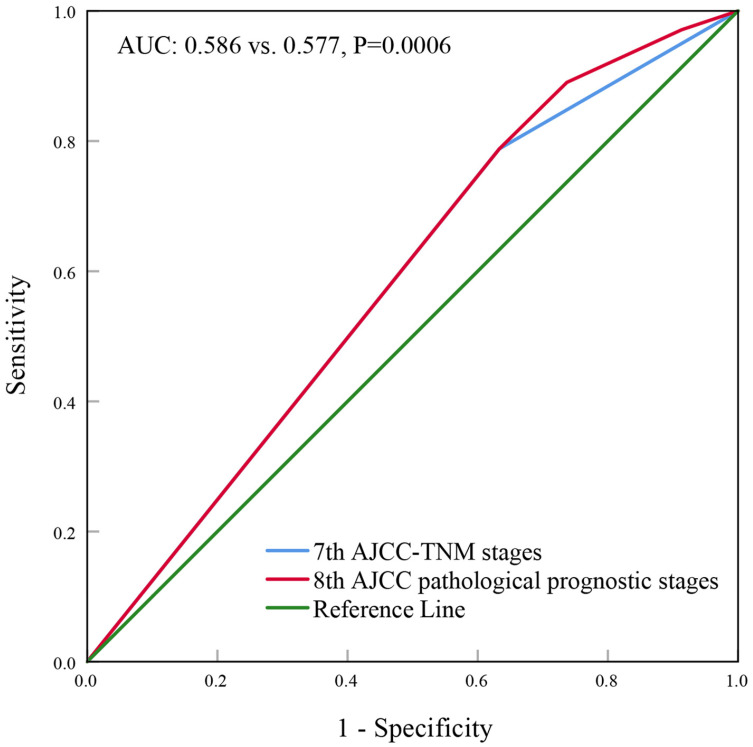
Receiver operating characteristics analyses for predicting the breast cancer-specific survival between the 7th and 8th AJCC staging system.

With a median follow up of 41.5 (range, 0-83) months, the 5-years BCSS rate of the 7th AJCC stage IIIC and IV patients was 58.4% and 39.1%, respectively (Figure 1). The new pathological prognostic staging system has re-stratified these ISLN-metastasized BC patients, with a 5-year BCSS rate of 71.3%, 62.2%, 45.2% and 39.1% in stage IIIA, IIIB, IIIC, and IV cohorts, respectively (*P*<0.0001) (Figure 2). The ROC analysis demonstrated that the AJCC 8th edition pathological prognostic staging had superior discriminative ability than the 7th AJCC-TNM staging in predicting the BCSS (AUC: 0.586 vs. 0.577, *P*=0.0006) (Figure 3).

### Multivariate prognostic analysis

Two prognostic models were conducted to assess the independent prognostic factors associated with BCSS. The first Cox proportional hazard model was incorporating ER, PR, HER2 status, and tumor grade with other demographic and clinical characteristics, the results that age, race/ethnicity, tumor grade, T category, M stage, ER, PR, and HER2 status were the independent prognostic factors related to BCSS (Table 3). In the second model, the 8th AJCC pathological prognostic staging was included in the multivariate prognostic analysis. The results indicated that the AJCC 8th edition pathological prognostic stage was the independent prognostic factor for BCSS. However, the stage IIIC disease showed comparable BCSS compared with the stage IV disease (hazard ratio [HR]=0.820, 95% confidence interval [CI]: 0.611-1.102,* P*=0.188) (Table 4).

## Discussion

Unlike those previous staging systems for breast cancer, the newly revised 8th AJCC staging included not only TNM factors but also four important biological factors (ER status, PR status, HER2 status, and tumor grade) 5. Several studies have confirmed that the 8th AJCC staging could more accurately predict the prognosis of BC patients 7-16. However, to our knowledge, no studies assessed the prognosis accuracy of the new AJCC staging for patients with ISLN metastasis 7-16. Our study was the first study to evaluate the role of the AJCC 8th edition of pathological prognostic staging systems in ISLN-metastasized BC patients.

In the newly revised staging system, the ISLN-metastasized BC without distant metastasis was no longer generally assigned into stage IIIC but would be stratified into stage IIIA, IIIB, or IIIC according to the biological characteristics. In our study, 64.9% of the ISLN-metastasized patients (7th AJCC-TNM stage IIIC) were reassigned and downstaged to stage IIIA or IIIB using the 8th AJCC criteria. Overall, the discriminatory performance of the AJCC 8th edition of pathological prognostic staging in ISLN-metastasized BC patients was superior to the 7th AJCC-TNM staging. The multivariate prognostic analysis revealed that the AJCC 8th edition of the pathological prognostic stage was an independent prognostic factor for BCSS, but there was no statistical difference in BCSS between the 8th AJCC stage IIIC and IV patients.

With the evolving knowledge of breast cancer biology, biological factors are gradually elevated to similar importance in the determination of AJCC staging. It was well acknowledged that ER, PR, and HER2 status were associated with the prognosis of BC patients and were regarded as predictive indicators of benefit from endocrine or anti-HER2 therapy 17. In our study, ISLN-metastasized patients with ER-positive or PR-positive diseases were associated with better survival outcomes. Moreover, we found that 86.6% and 97.1% of patients with ER-positive and PR-positive were downstaged, demonstrating that those ER-positive or PR-positive patients tend to be associated with lower stage and have a better prognosis in comparison with ER-negative and PR-negative tumors 18-21. Another study from Lee *et al.* 22 found that the prognosis of stage III patients with hormone receptor (HR)+/HER2- disease was better than those of stage II patients with HR-/HER2- diseases. In our study, the rate of downstaging in HER2-positive patients was higher than that in HER2-negative patients (100.0% vs. 46.9%) after using the novel staging system, and the multivariate analysis also showed that HER2-positive disease was a favorable prognostic factor for BCSS compared to those with HER2-negative disease. Thus, HER2-positive status instead of HER2-negative status should be deemed as a better prognostic factor in patients who received anti-HER2 therapy. Howlader* et al.* 23 also reported that HR+/HER2+ subtype had better survival than HR+/HER2- in advanced-stage BC in the era of anti-HER2 targeted therapy.

The role of ISLN metastasis in the BC staging system has undergone two significant changes. The first change occurred in 1988, ISLN-metastasized BC without distant metastasis was allocated into stage M1 from stage IIIB. The second change occurred in 2002, ISLN-metastasized BC was reclassified into stage IIIC from stage M1 in the 6th AJCC staging system. Different studies on the prognosis of ISLM-metastasized BC patients have raised conflict results 3, 24-32. Several studies showed that BC with ISLN metastasis had a better survival outcome than that with distant metastasis disease, and was potentially curable 3, 4, 24-28. However, a study reported that the prognosis of ISLN-metastasized BC was better than stage IV but worse than stage IIIB and IIIC 29. With the development of BC molecular biology, researchers revealed that ISLN-metastasized BC was a disease of significant heterogeneity with significantly different long-term prognosis.

In the 8th AJCC staging, most of the patients diagnosed with stage IIIC disease were with G3 and triple-negative disease (ER-negative, PR-negative, and HER2-negative), which were relevant to a significantly higher risk of distant metastasis and worse survival. Our results showed comparable BCSS in patients with stage IIIC disease and M1 disease, which revealed that the 8th AJCC stage IIIC ISLN-metastasized BC might be reclassified into stage M1.

In our study, we found that 70.6% of the ISLN-metastasized BC patients developed distant metastasis at the time of diagnosis. ISLN metastasis might be a potentially high-risk factor for distant metastasis. The 5-year distant metastasis rates of BC patients with ISLN metastasis were reported to be 77.8-85.5% 25, 32, 33. The 2-year distant metastasis rate of ISLN-metastasized patients in the study from Fan *et al.* 32 was 60.4%, which meant that patients with ISLN metastasis had a significantly high risk of distant metastasis. Furthermore, there was a higher proportion of triple-negative breast cancer (TNBC) in BC patients with ISLN metastasis, compared with other stage N3 patients without ISLN metastasis 34, 35. TNBC was a unique subtype in BC that was unable to benefit from endocrine therapy and anti-HER2 therapy. These factors stated above may explain why although most stage M0 patients received a comprehensive treatment including surgery (100.0%), chemotherapy (93.5%) and radiotherapy (73.6%) in our study, there was still no significant statistical difference in the 5-year BCSS rates between the 8th AJCC stage IIIC patients and stage M1 patients. In recent years, multimodality treatment including surgery, radiotherapy, chemotherapy, endocrine therapy, and targeted therapy with curative intent has been strongly recommended for BC patients with ISLN metastasis 3, 24, 27, 29, 36, 37. Taken together, we need to explore more new therapeutic targets to improve the survival of ISLN-metastasized patients.

We acknowledged that several limitations were exiting in this study. Firstly, as a retrospective study, the inherent defects are inevitable in our study. Secondly, the local relapse and metastatic pattern of the ISLN-metastasized patients were unknown in our study. Thirdly, there was no record on endocrine and anti-HER2 therapy in the SEER database. However, our study was in the context of contemporary treatment, and most patients received chemotherapy. Therefore, we could assume that most patients in our study also received corresponding multidisciplinary therapy. Finally, the duration of follow up in our study was relatively short; studies with a more extended period of follow up are needed to confirm our results.

## Conclusion

In conclusion, our study suggests that ISLN-metastasized BC is a disease entity of significant heterogeneity. Compared with the 7th AJCC staging, the 8th AJCC pathological prognostic staging showed a better discriminatory value of prognosis among ISLN-metastasized BC patients. An additional clarification strategy in stage IIIC disease based on the 8th AJCC staging should be developed to differentiate patients who are curable with multimodality therapy and patients who have less benefit from curative treatment. Large sample, multi-center, and long-term follow up studies are required to verify our results.

## Figures and Tables

**Table 1:** Patient and tumor characteristics

Variables	n (%)	M0 (%)	M1 (%)
**Age (years)**			
<50	270 (24.6)	108 (33.5)	162 (20.9)
≥50	827 (75.4)	214 (66.5)	613 (79.1)
**Race/ethnicity**			
Non-Hispanic White	626 (57.1)	190 (59.0)	436 (56.2)
Non-Hispanic Black	225 (20.5)	56 (17.4)	169 (21.8)
Hispanic (All Races)	157 (14.3)	55 (17.1)	102 (13.2)
Other	89 (8.1)	21 (6.5)	68 (8.8)
**Histological subtype**			
Infiltrating ductal carcinoma	796 (72.6)	254 (78.9)	542 (69.9)
Invasive lobular carcinoma	70 (6.4)	19 (5.9)	51 (6.6)
Other	231 (21.0)	49 (15.2)	182 (23.5)
**T category**			
T1	91 (8.3)	39 (12.1)	52 (6.7)
T2	262 (23.9)	120 (37.3)	142 (18.3)
T3	177 (16.1)	62 (19.2)	115 (14.8)
T4	502 (45.8)	101 (31.4)	401 (51.7)
Tx	65 (5.9)	0 (0)	65 (8.4)
**Grade**			
Well differentiated	32 (2.9)	8 (2.5)	24 (3.1)
Moderately differentiated	292 (26.6)	94 (29.2)	198 (25.5)
Poorly/undifferentiated	625 (57.0)	220 (68.3)	405 (52.3)
Unknown	148 (13.5)	0 (0)	148 (19.1)
**ER status**			
Negative	409 (37.3)	136 (42.2)	273 (35.2)
Positive	657 (59.9)	186 (57.8)	471 (60.8)
Unknown	31 (2.8)	0 (0)	31 (4.0)
**PR status**			
Negative	559 (51.0)	185 (57.5)	374 (48.3)
Positive	496 (45.2)	137 (42.5)	359 (46.3)
Unknown	42 (3.8)	0 (0)	42 (5.4)
**HER2 status**			
Negative	721 (65.7)	213 (66.1)	508 (65.6)
Positive	313 (28.5)	109 (33.9)	204 (26.3)
Unknown	63 (5.7)	0 (0)	63 (8.1)
**Surgery**			
No surgery	469 (42.8)	0 (0)	469 (60.5)
BCS	145 (13.2)	69 (21.4)	76 (9.8)
MAST	483 (44.0)	253 (78.6)	230 (29.7)
**Radiotherapy**			
No	581 (53.0)	85 (26.4)	496 (64.0)
Yes	516 (47.0)	237 (73.6)	279 (36.0)
**Chemotherapy**			
No	305 (27.8)	21 (6.5)	284 (36.6)
Yes	792 (72.2 )	301 (93.5)	491 (63.4)

T, tumor; M, distant metastasis; ER, estrogen receptor; PR, progesterone receptor; HER2, human epidermal growth factor receptor 2; BCS, breast-conservation surgery; MAST, mastectomy.

**Table 2:** Comparisons of demographic and tumor characteristics among stage change from the 7th to the 8th edition of the AJCC breast cancer staging system (stage M0 patients)

Variables	Downstage (%)	No change (%)	*P*
**Age (years)**			
<50	58 (53.7)	50 (46.3)	0.003
≥50	151 (70.6)	63 (29.4)	
**Race/ethnicity**			
Non-Hispanic White	130 (68.4)	60 (31.6)	0.016
Non-Hispanic Black	29 (51.8)	27 (48.2)	
Hispanic (All Races)	32 (58.2)	23(41.8)	
Other	18 (85.7)	3 (14.3)	
**Histological subtype**			
Infiltrating ductal carcinoma	160 (63.0)	94 (37.0)	0.162
Invasive lobular carcinoma	16 (84.2)	3 (15.8)	
Other	33 (67.3)	16 (32.7)	
**T category**			
T1	23 (59.0)	16 (41.0)	0.874
T2	79 (65.8)	41 (34.2)	
T3	41 (66.1)	21 (33.9)	
T4	66 (65.3)	35(34.7)	
**Grade**			
Well differentiated	8 (100.0)	0 (0)	<0.001
Moderately differentiated	82 (87.2)	12 (12.8)	
Poorly/undifferentiated	119 (54.1)	101 (45.9)	
**ER status**			
Negative	48 (35.3)	88 (64.7)	
Positive	161 (86.6)	25 (13.4)	<0.001
**PR status**			
Negative	76 (41.1)	109 (58.9)	
Positive	133 (97.1)	4 (2.9)	<0.001
**HER2 status**			
Negative	100(46.9)	113 (53.1)	
Positive	109(100.0)	0 (0)	<0.001

T, tumor; M, distant metastasis; ER, estrogen receptor; PR, progesterone receptor; HER2, human epidermal growth factor receptor 2.

**Table 3:** Multivariate analysis of prognostic factors using the Cox- regression model (including biologic factors)

Variables	HR	95%CI	*P*
**Age (years)**			
<50	1		
≥50	1.345	1.087-1.665	0.006
**Race/ethnicity**			
Non-Hispanic White	1		
Non-Hispanic Black	1.419	1.140-1.766	0.002
Hispanic (All Races)	1.075	0.813-1.421	0.613
Other	0.917	0.656-1.283	0.613
**Histological subtype**			
Infiltrating ductal carcinoma	1		
Invasive lobular carcinoma	1.342	0.931-1.935	0.115
Other	1.048	0.833-1.319	0.688
**T category**			
T1	1		
T2	0.984	0.670-1.445	0.933
T3	1.106	0.741-1.652	0.621
T4	1.574	1.097-2.258	0.014
Tx	1.917	1.134-3.242	0.015
**M category**			
M0	1		
M1	2.570	2.055-3.214	<0.001
**Histological grade**			
Well differentiated	1		
Moderately differentiated	1.185	0.677-2.072	0.553
Poorly/undifferentiated	1.385	0.797-2.406	0.248
Unknown	0.244	0.128-0.464	<0.001
**ER status**			
Negative	1		
Positive	0.661	0.517-0.845	0.001
Unknown	0.825	0.309-2.203	0.701
**PR status**			
Negative	1		
Positive	0.680	0.532-0.869	0.002
Unknown	1.275	0.579-2.805	0.546
**HER2 status**			
Negative	1		
Positive	0.531	0.429-0.657	<0.001
Unknown	0.752	0.476-1.188	0.222

HR, hazard ratio; CI, confidence interval; T, tumor; M, distant metastasis; ER, estrogen receptor; PR, progesterone receptor; HER2, human epidermal growth factor receptor 2.

**Table 4:** Multivariate analysis of prognostic factors using the Cox-regression model (including 8th AJCC pathological prognostic stages)

Variables	HR	95%CI	*P*
**Age (years)**			
<50	1		
≥50	1.112	0.900-1.374	0.324
**Race/ethnicity**			
Non-Hispanic White	1		
Non-Hispanic Black	1.299	1.051-1.606	0.015
Hispanic (All Races)	0.848	0.644-1.117	0.241
Other	0.906	0.650-1.264	0.562
**Histological subtype**			
Infiltrating ductal carcinoma	1		
Invasive lobular carcinoma	1.155	0.815-1.637	0.417
Other	0.795	0.795-0.637	0.044
**T category**			
T1	1		
T2	1.074	0.732-1.576	0.716
T3	1.262	0.848-1.881	0.254
T4	1.663	1.162-2.381	0.005
Tx	1.246	0.746-2.082	0.401
**8th AJCC pathological prognostic stages**			
IV	1		
IIIA	0.312	0.186-0.525	<0.001
IIIB	0.415	0.300-0.573	<0.001
IIIC	0.820	0.611-1.102	0.188

HR, hazard ratio; CI, confidence interval.
